# METTL3 alleviates renal tubular mitochondrial dysfunction by regulating the TUG1/PGC-1a axis in an IGF2BP2-dependent manner in diabetic nephropathy

**DOI:** 10.1080/0886022X.2025.2521455

**Published:** 2025-07-20

**Authors:** Tong Chen, Juan Wang, Yanyan Xu, Yonghong Zhu, Ying Jin, Qiuling Fan

**Affiliations:** a Department of Nephrology, Shenyang Seventh People’s Hospital, Shenyang, Liaoning, China; b Department of Nephrology, Shanghai General Hospital, Shanghai Jiao Tong University School of Medicine, Shanghai, China; c Department of Nephrology, The Fourth Affiliated Hospital of China Medical University, Shenyang, Liaoning, China; d Department of Nephrology, The First Affiliated Hospital of China Medical University, Shenyang, Liaoning, China; e Department of Acupuncture, Liaoning University of Traditional Chinese Medicine, Shenyang, Liaoning, China

**Keywords:** Diabetic nephropathy, m6A modification, METTL3, lncRNA TUG1, mitochondria

## Abstract

**Purpose:**

To explore the N6-methyladenosine (m^6^A) modification mechanism of taurine upregulated gene 1 (TUG1) and whether methyltransferase 3 (METTL3) can promote peroxisome proliferators-activated receptor γ coactivator 1 alpha (PGC-1α) transcription and alleviate mitochondrial dysfunction.

**Methods:**

*In vitro* high glucose (HG)-treated HK-2 cell models and *in vivo* db/db mice models injected with rAAV-METTL3 *via* the tail vein were established. The expression levels were determined by RT–qPCR, western blot, and immunohistochemical staining. RNA m^6^A modification was analyzed by the RNase Mazf. The biochemical indicators of mice were detected by enzyme-linked immunosorbent assay. Cell apoptosis was detected by flow cytometry. Histopathological staining was performed to evaluate kidney injury. mtDNA content, mitochondrial complex activity, and ATP were detected by RT–qPCR and detection kits, respectively, per the manufacturer’s instructions. Mitochondrial reactive oxygen species production in HK-2 cells incubated with MitoSOX Red and mitochondrial morphology were observed under a fluorescence microscope and transmission electron microscope, respectively. Molecular interactions were verified through RNA immunoprecipitation, RNA pull-down, and dual-luciferase reporter gene assay.

**Results:**

METTL3 and TUG1 expression levels decreased in the kidneys of diabetic mice and HG-treated HK-2 cells. Mechanistically, METTL3-mediated m^6^A modification increased the stability of TUG1 in an insulin-like growth factor 2 mRNA binding protein 2 (IGF2BP2)-dependent manner. METTL3-mediated m^6^A modification of TUG1 promotes PGC-1α activation, thereby alleviating mitochondrial dysfunction in HG-treated HK-2 cells and db/db mice. Moreover, METTL3 overexpression alleviated kidney injury in db/db mice.

**Conclusion:**

METTL3 targets TUG1/PGC-1α and ameliorates mitochondrial dysfunction in diabetic nephropathy in an IGF2BP2-dependent manner.

## 1. Introduction

DN is one of the most common and serious chronic microvascular complications of diabetes [1]. In the context of DN, in addition to hyperglycemia, advanced glycation end products (AGEs), oxidative stress, chronic continuous inflammation, and lipid metabolic disorders can coordinate to promote epigenetic changes and mitochondrial dysfunction [2–8]. In renal tubular cells with abundant mitochondria, oxidative stress caused by persistent hyperglycemia can lead to an imbalance in oxidative and antioxidative systems, leading to mitochondrial damage and eventually renal tubular injury [9]. Epigenetic modifications participate in the development of DN by regulating gene transcription and translation [10]. In this regard, epigenetic markers that improve mitochondrial dysfunction can help us stratify patients at risk for diabetic kidney disease (DKD).

Increasing research on RNA epigenetics has revealed m^6^A as the most abundant internal RNA modification, which was first discovered in the 1970s and had been shown to play key roles in noncoding RNA (ncRNA) [11,12]. The distribution of m^6^A exhibits tissue specificity, with the kidney having one of the highest distributions of m^6^A [13]. m^6^A is catalyzed by the “writer”-methyltransferase complex including METTL3, methyltransferase 14 (METTL14), and Wilms Tumor 1 associated protein (WTAP) on the consensus motif “RRACH” (R = G/A; H = A/C/U) [14]. In contrast, the ‘erasers’ demethylases including alkB homolog 5 (ALKBH5) and fat mass and obesity associated (FTO) can remove m^6^A from RNA [15]. The ‘reader’ modification recognition protein can function as the RNA binding proteins (RBPs) allowing the methylated RNA to be functionalized, including the YTH domain family (YTHDF) and IGF2BPs [16]. YTHDF regulates RNA stability, while IGF2BPs improve the stability and storage of their targeted RNAs in an m^6^A-dependent manner [17,18]. m^6^A modification participates not only in fat formation, obesity, islet β cell function, and glycometabolism but also in the occurrence and development of DN [18,19]. Wang et al. reported that METTL3 overexpression protected mouse renal tubular epithelial cells from colistin-induced kidney injury by modulating the Kelch-like ECH-associated protein 1 (Keap1)/nuclear respiratory factor 2 (Nrf2) pathway [20]. Jiang et al. reported that METTL3 aggravated podocyte injury in DN by regulating tissue inhibitor of metalloproteinases (TIMP2) mRNA in an m^6^A-dependent manner [21]. This controversial evidence suggests that the roles and underlying mechanisms of METTL3 in DN should be investigated.

Studies have shown that m^6^A modification not only plays a role in mRNA, but also regulates long noncoding RNAs (lncRNAs) by providing a binding site for m^6^A reader proteins or by adjusting the structure of local RNA to induce RBP entry [22]. Among all lncRNAs related to kidney disease, TUG1 is of particular interest. TUG1 plays important roles in many human kidney diseases, such as DN, acute kidney injury, lupus nephritis, interstitial fibrosis, and renal cell carcinoma. Accumulating evidence shows that TUG1 can inhibit cell apoptosis, endoplasmic reticulum stress (ERS), the release of inflammatory factors, and extracellular matrix (ECM) secretion, thereby protecting HK-2 cells from HG-induced damage [23–25]. A recent study revealed that METTL3 and TUG1 are downregulated in the testicular tissues of diabetic mice and HG-treated GC-1 spermatogonial (spg) cells [26]. Mechanistically, METTL3-mediated m^6^A methylation enhances the stability of TUG1, and inhibition of TUG1/clusterin signaling markedly reverses the protective effect of METTL3 overexpression on HG-stimulated GC-1 spg cells [26]. However, due to the tissue specificity of m^6^A, how METTL3 modifies TUG1 in DN and the relationship between METTL3-mediated m^6^A modification of TUG1 and DN progression have not been fully elucidated. These questions deserve further investigation.

The pattern of m^6^A is more important than the quantity, and a low level of m^6^A located in specific patterns on the RNA can greatly impact the outcome for that RNA. Therefore, RNA sequencing could better reveal the effects of m^6^A modification level. Our research group used an Arraystar m^6^A single-base resolution chip to detect m^6^A expression in human renal tubular epithelial cells and discovered a significant decrease in the m^6^A level of 35 noncoding RNAs under HG conditions. There was a significant difference in the abundance of m^6^A modifications in lncRNA TUG1, located on chromosome 22 in HK-2 cells. Using the full-length sequence of the transcript, we identified four m^6^A sites in TUG1 in HK-2 cells (Supplemental Figure 1) and two identical m^6^A sites in TUG1 on chromosome 11 corresponding to mice (Supplemental Table 2). Overall, we focused on the m^6^A modification mechanism of TUG1 and investigated how modified TUG1 improves mitochondrial dysfunction in DN, hoping to provide a theoretical basis for new therapeutic strategies.

## 2. Materials and methods

### 2.1. Animal model

All animal experiments were approved by Animal Ethical Committee of China Medical University (IACUC Issue No.2020312). A total of 11 male db/m (C57BLKS/J-leprdb/+) mice (7-8 weeks old) and 12 db/db (C57BLKS/J-leprdb/leprdb) mice were acquired from the Model Animal Research Center of Nanjing University. Recombinant Adeno-Associated virus (rAAV) 2/9-METTL3 was purchased from Sangon Biotechnology (Shanghai, China). The experimental mice were maintained under standard conditions. All animals were housed at a 22 °C constant room temperature and 47% humidity with a 12-h light–dark cycle and free access to standard laboratory chow and tap water. After acclimatization for 1 week, the mice were randomly separated into 3 groups (*n* = 6): the db/m, db/db, and db/db + rAAV-METTL3 groups. First, 5 db/m mice were used to verify the concentration and transfection efficiency of rAAV. The third group was injected with rAAV to deliver METTL3 *via* the tail vein. A total of 100 µl of virus (1 × 10^11^) was injected into the tail vein using an insulin needle and maintained for 2-3 s. The blood glucose and body weight of the mice were monitored weekly. Urine samples were collected from metabolic cages of the mice every 4 weeks for determination of the urine albumin/creatinine ratio. Twelve weeks after injection, 20-week-old mice were sacrificed, and kidney tissues and blood samples were collected for further analysis. Blood was collected to determine blood urea nitrogen (BUN), plasma albumin (ALB), and creatinine levels. The kidneys were collected for paraffin embedding, electron microscopy observation in specific fixation fluid, and molecular biological analysis. The experimental mice were euthanized humanely after the administration of anesthesia.

### 2.2. Detection of blood glucose levels

After the mice were fasted for 12 h, blood samples were collected, and the blood glucose concentration was measured with a glucometer (Yuyue580, Jiangsu Yuyue Medical Equipment Company, China).

### 2.3. Detection of BUN, ALB, and creatinine

BUN, ALB, and creatinine were measured using BUN, ALB, and creatinine determination kits (Bioengineering Research Institute of Nanjing Jiancheng Biological Company) by ELISA through a microplate reader (Shanghai Flash Spectrum Biotechnology, China) following the manufacturer’s instructions.

### 2.4. Cell culture

Human renal proximal tubular epithelial cell line (HK-2) purchased from American Type Culture Collection (ATCC, USA) was maintained in Dulbecco’s Modified Eagle’s Medium/Nutrient Mixture F-12 (DMEM/F12) (Gibco, Australia) medium supplemented with 5.5 mmol/L glucose and 10% fetal bovine serum (FBS, Gibco, Australia) at 37 °C with 5% CO2. To establish a disease model *in vitro*, HK-2 cells were incubated with D-glucose (5.5, 10, 15, 20, 25, 30, 40 mM) for up to 72 h. Then, according to cell counting kit-8 assay (Supplemental Methods and Figure 2), 25 mM D-glucose as the HG group and 5.5 mM D-glucose as the normal glucose (NG) group were chosen as the appropriate concentration for further experiments.

### 2.5. Plasmid construction and cell transfection

The sequence of wild-type METTL3 was amplified and cloned and inserted into the pcDNA 3.1 vector (Invitrogen, USA) to construct METTL3 and IGF2BP2 expression plasmids. Specific siRNAs against TUG1 were designed and synthesized by Generaybiotech (Beijing, China). When HK-2 cells reached 80% confluence, they were transfected with plasmids or siRNAs using Lipofectamine (Invitrogen, USA) according to the manufacturer’s instructions.

### 2.6. Western blot

Kidney tissues and HK-2 cells were lysed in RIPA lysis buffer (GenStar, China) and the total protein content was determined by with a BCA kit (GenStar, China). The primary antibodies from Proteintech Group (USA) used were as follows: Anti-METTL3 (1:1000), Anti-METTL14 (1:1000), Anti-FTO (1:1000), Anti-YTHDF1 (1:5000), Anti-YTHDF2 (1:5000), Anti-IGF2BP2 (1:2000), Anti-PGC-1α (1:1000), Anti-(nuclear respiratory factor 1) Nrf1 (1:2000), Anti-Nrf2 (1:1000), Anti-(mitochondrial transcription factor A) TFAM (1:2000), and Anti-β-actin (1:5000). After incubation with an HRP-conjugated secondary antibody, the protein bands were visualized using an enhanced chemiluminescence (ECL) kit (Shanghai Bioscience Technology Company, China) and analyzed using a Tanon-4500 Gel Imaging System (Tanon, China).

### 2.7. Real-time quantitative polymerase chain reaction (RT-qPCR)

Total RNA from renal tissues and HK-2 cells was extracted with TRIzol Reagent (GenStar, China) and then reverse transcribed with a First-Strand cDNA Synthesis Kit (GenStar, China). RT–qPCR was performed with METTL3, METTL14, FTO, YTHDF1, YTHDF2, IGF2BP2, PGC-1α, Nrf1, Nrf2, TFAM, β-actin, and TUG1 gene-specific primers from Genscript company (Nanjing) (Supplemental Table 3) and SYBR Green PCR Master Mix (GenStar, China). β-Actin was used as an endogenous control. The data were analyzed with the 2^–ΔΔCt^ method.

### 2.8. Measurement of m^6^A modification

RNA was extracted from HK-2 cells and renal samples from db/m mice and db/db mice using TRIzol reagent (GenStar, China), as recommended by the manufacturer. A NanoDrop (Thermo Fisher Scientific, MA, USA) was used to analyze the RNA quality. After standardizing the sample quality, the overall m^6^A levels of RNA were evaluated using the EpiQuik m^6^A RNA methylation quantification kit (colorimetric method, EpigenTek, P-9005-48) according to the manufacturer’s instructions. The total content of m^6^A was calculated using the following formula: m^6^A% = (Sample OD − NC OD)/Slope/*S* × 100%, where OD, NC, S, PC, and P represent the optical density, negative controls, total amount of input RNA, positive controls, and total amount of positive control RNA, respectively.

### 2.9. RNase MazF

Cellular RNAs were digested at the unmethylated ACA site using the bacterial single-stranded RNase MazF. Sites with m^6^A methylation remain uncleaved. Given MazF’s ability to discriminate between 5′-ACA-3′ and 5′-(m^6^A)CA-3′, we determined the m^6^A methylation site on TUG1. When performing RT-qPCR with specific primers (Supplemental Table 4), 1 μl of each sample was added, and MazF- was used as a control. The MazF correction formula was as follows: % MazF- = (2^–CtMazF +^)/(2^–CtMazF –^)×100%.

### 2.10. IHC staining

For IHC studies, paraffin-embedded kidney tissues were deparaffinized and hydrated using slide warmers and alcohol. After antigen retrieval, the sections were permeabilized with 3% H2O2 and blocked with 5% bovine serum albumin (BSA). Then, the sections were individually incubated with the following antibodies at the appropriate concentrations: antibodies against METTL3 (1:200), IGF2BP2 (1:200), Nrf1 (1:200), Nrf2 (1:200), and TFAM (1:200) from Proteintech. Then, the sections were incubated with secondary antibodies and reacted with diaminobenzidine (DAB) according to the manufacturer’s instructions.

### 2.11. Histopathological evaluation

Kidney sections were stained with hematoxylin and eosin (HE), Masson and periodic acid-Schiff (PAS) to assess kidney injury. Briefly, renal tissues from each mouse were fixed in 4% paraformaldehyde, embedded in paraffin, and then sectioned at a thickness of 4 μm. Finally, the sections were mounted in neutral balsam for microscopic observation. HE staining (Abcam, UK) was performed to detect general morphological changes, the glomerular area, and the PAS-stained positive matrix area to determine the ratio of the matrix-positive area. Masson staining (Sigma-Aldrich, USA) was used to assess matrix deposition within the interstitium according to standard protocols. Tubulointerstitial impairment was evaluated according to the scoring criteria as follows: 0 = none; 1 = mild or <25%; 2 = moderate or 25% to 50%; and 3 = severe or >50%. The blue Masson staining area was used to indicate collagen deposition. Six nonoverlapping fields in each section of the kidney were selected for scoring and image analysis using Image J, and the results are expressed as mean ± SD.

### 2.12. Measurement of mitochondrial DNA (mtDNA) content

DNA was isolated from HK-2 cells and mouse kidney tissues using a QIAamp DNeasy Blood and Tissue Kit (Qiagen). The quality and concentration of the isolated DNA were assessed using a Nanodrop 1000 spectrophotometer (Shanghai Flash Biotech Company, China). The relative mtDNA content in HK-2 cells was measured *via* RT-qPCR by determining the ratio of two mitochondrial gene copy numbers, mitochondrial forward primer from nucleotide 3212/reverse primer from nucleotide 3319 (MTF3212/R3319) and mitochondrial encoded NADH dehydrogenase 1 (MT-ND1), to that of the single-copy nuclear control gene acidic ribosomal phosphoprotein (RPLP0). For mouse kidney tissues, RT-qPCR was used to determine the ratio of two mitochondrial gene copy numbers, MT-CytB and MT-ND1, to a single copy of the nuclear control gene, MT-H19 (Supplemental Table 2). Each PCR was conducted in triplicate, and three non-template controls and six inter-run calibrators were included in each 96-well plate. All samples were analyzed using an RT-PCR system. Following thermal cycling, the raw data were acquired and subsequently processed. The cycle threshold (CT) values corresponding to the two mitochondrial genes were standardized in relation to a nuclear reference gene by qBase software (Biogazelle, Zwijnaarde, Belgium).

### 2.13. Mitochondrial complex activity and adenosine triphosphate(ATP) determination

Cellular ATP levels were measured using an ATP content detection kit (Solarbio, Beijing, China) per the manufacturer’s instructions. The activities of cellular mitochondrial complexes I, II, III, and IV were measured using a mitochondrial complex activity detection kit (Solarbio, Beijing, China) per the manufacturer’s instructions.

### 2.14. Examination of mitochondrial morphological changes by TEM and fluorescence staining

The mitochondria in HK-2 cells and renal tubules of kidney tissues were observed by TEM (Philips, Electron Optics) at magnifications of 1000 and 2000 ×.

### 2.15. m^6^A RIP-PCR

A Magna RIP Kit (Millipore, MA, USA) was used to perform RIP. Firstly, HK-2 cells were washed with pre-cooled PBS and gently scraped off the culture plate. Then, RIP analysis was performed with an RBP collected by centrifugation at 1500 rpm for 5 min at 4 °C. The RIPA lysis buffer was added to lyse the cells. The magnetic beads were incubated with anti-METTL3 and anti-IGF2BP2 antibodies (Proteintech Group, USA) for 30 min at room temperature, carefully mixed with lysis buffer, and incubated overnight at 4 °C. The protein-RNA complex was digested with proteinase K to purify the RNA, then, RNA was extracted with phenol:chloroform:isoamyl alcohol (125:24:1) (Solarbio, Beijing, China) and subjected to reverse transcription. Finally, lncRNA TUG1 was analyzed *via* RT-qPCR. The method used for RNA m^6^A immunoprecipitation was similar to that used for METTL3 and IGF2BP2. After digestion with DNase I, the RNA was subjected to a 10-s sonication process to induce fragmentation. Magnetic beads were subjected to incubation with rabbit anti-m^6^A antibody (Proteintech Group, USA) at room temperature for 60 min, and then the mixture of fragmented RNA, antibody-magnetic bead complex, and RIP buffer were incubated overnight at 4 °C. After the proteinase K buffer was utilized to digest the complex, RNA was then extracted using a phenol:chloroform:isoamyl alcohol (125:24:1) solution, Subsequent to this purification step, reverse transcription and RT-qPCR were carried out with specific primers (Supplemental Table 4) to quantify the presence of lncRNA TUG1. IgG antibody (Proteintech Group, USA) was employed as a negative control.

### 2.16. RNA pull-down assay

BersinBio RNA pull-down kit (BersinBio, Guangzhou, China) was used to performed RNA pulldown. The biotin-labeled lncRNA-TUG1 was designed and synthesized by Generaybiotech (Beijing, China). Cell lysate was prepared in RIP buffer and then mixed with biotin-labeled lncRNA TUG1 RNAs incubated at 4 °C for 1 h. Subsequently, the complex was separated by streptavidin conjugated magnetic beads. Finally, the recovered proteins were identified using Western blot to measure METTL3 (Proteintech Group, USA) level.

### 2.17. Measurement of RNA stability

To measure the half-life of TUG1, we added the transcriptional inhibitor actinomycin D (Act D, 5 μg/mL; Saitong, China) to the culture medium of transfected HK-2 cells for 30 min. Total RNA was collected at 0, 1, 3, 6, 9, and 12 h and subjected to RT-qPCR analysis. The relative expression level of the transcript was normalized to that of β-actin.

### 2.18. Dual-luciferase reporter gene assay

To detect TUG1/PGC-1α interaction in cultured cells, HK-2 cells were transfected with empty vector pGL3, Flag-PGC1α, METTL3 overexpression plasmids or siTUG1 using Lipofectamine (Invitrogen, USA) according to the manufacturer’s protocols. Luciferase activity was measured using the dual-luciferase reporter kit (Promega, USA) after 48 h of incubation. Finally, relative luciferase activity was normalized to the renilla and quantified by the luminescence plate reader (SuPerMax 3100, Shanghai).

### 2.19. Detection of intracellular reactive oxygen species (ROS) production

To assess intracellular ROS production, we incubated the cells with MitoSOX Red (5 μM) (Yeasen, Shanghai, China) in serum-free culture medium at 37 °C for 20 min. Mitochondrial ROS production in kidney tissues was assessed using 4-μm-thick frozen sections, which were subsequently stained with MitoSOX. After three washes with PBS, the cells were observed under a fluorescence microscope. ImageJ software was used to measure the fluorescence intensity of MitoSOX and the density per area.

### 2.20. Apoptosis assay

The degree of tubular cell apoptosis *in vitro* was assessed by flow cytometry with Annexin V-FITC/PI double staining per the manufacturer’s instructions (Annexin V-FITC/PI Kit, Biotech, China).

### 2.21. Statistical analysis

All the statistical analyses were performed using GraphPad software (USA). The data are presented as mean ± SD. Normally distributed data were analyzed by unpaired two-tailed Student’s t tests. Multiple groups were analyzed by one-way analysis of variance (ANOVA). If the data were not normally distributed, nonparametric tests were used. A P-value <0.05 was considered to indicate significance.

## 3. Results

### 3.1. METTL3, TUG1 and m^6^A modifications decreased in HG-induced HK-2 cells

**Figure 1.**
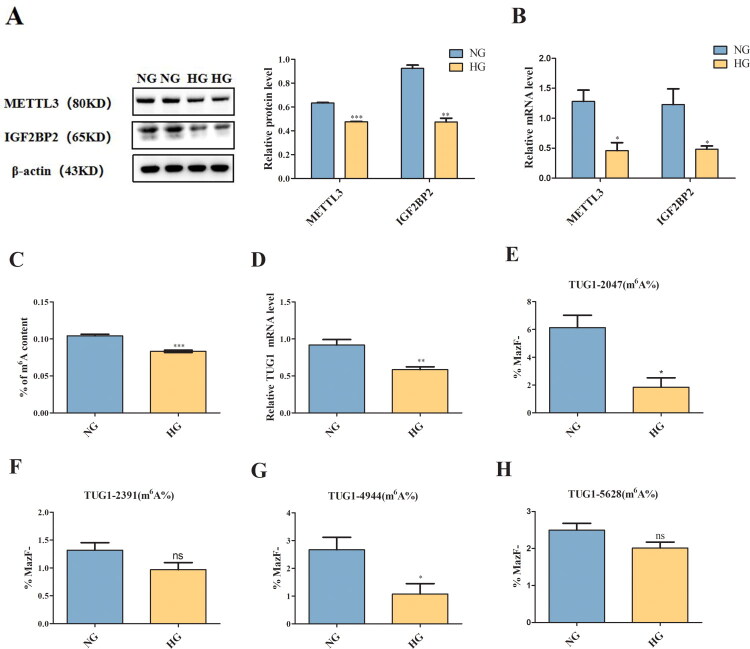
Levels of METTL3, TUG1 and its m^6^A modification in HK-2 cells. (A,B) METTL3 and IGF2BP2 expression levels in HK-2 cells were examined by Western blot and RT-qPCR (*n* = 5). (C) The m^6^A content of HK-2 cells was examined with an m^6^A RNA methylation quantitative detection kit (*n* = 3). (D) Relative expression of TUG1 mRNA in HK-2 cells were examined by RT-qPCR (*n* = 5). (E-H) RNase MazF-RT-qPCR method was used to detect the level of m^6^A modification of the four m^6^A sites of TUG1 respectively (*n* = 3). Data were presented as the mean ± SD. *p < 0.05, **p < 0.01, ***p < 0.001vs. NG, ns no statistically significant vs. NG.

*In vitro*, compared with those in the NG group, METTL3 and IGF2BP2 expression levels in HG-induced HK-2 cells significantly decreased at both the protein and mRNA levels (Figure 1(A,B)). Other regulators were not significantly different (Supplemental Figure 3). We also observed that the total m^6^A content and TUG1 expression significantly decreased in the HG group than in the NG group (Figure 1(C,D)), and only the m^6^A modification levels at sites 2047 and 4944 of TUG1 significantly decreased in HG-induced HK-2 cells (Figure 1(E–H)).

### 3.2. m^6^A modification increased the stability of TUG1 in HK-2 cells

We next investigated the mechanisms by which METTL3 regulates the stability of TUG1 in HK-2 cells under HG conditions. RIP-PCR analysis and RNA pull-down assay demonstrated that TUG1 could bind to METTL3, indicating that METTL3 could enhance the expression of TUG1 (Figure 2(A–D)). In addition, RIP-PCR analysis showed that IGF2BP2 can recognize m^6^A-modified TUG1 and increase the expression of TUG1 (Figure 2(E–G)). HG stimulation shortened the half-life of TUG1 and reduced its stability, whereas IGF2BP2 prolonged the half-life of TUG1 and improved its stability (Figure 2(H–J)), indicating that METTL3 could regulate TUG1 expression through the regulation of m^6^A modification in an IGF2BP2-dependent manner in HK-2 cells.

Figure 2.METTL3 Regulated TUG1 and its m^6^A modification in an IGF2BP2-dependent manner in HK-2 cells. (A-D) The correlation between TUG1 and METTL3 was determined by RIP and RNA pull-down method. M: Marker; A, D, G: IgG negative control group, B, E, H: METTL3 antibody. C, F, I: input positive control. (E-G) IGF2BP2 can identify TUG1 through RIP-PCR method in HK-2 cells. B, E, H: IGF2BP2 antibody. *p < 0.05, **p < 0.01 vs. NG, #*p* < 0.05, ##*p* < 0.01 vs. HG. ns no statistically significant vs. NG. (H-J) After overexpression of IGF2BP2 in HK-2 cells, with 30 min of incubation using the medium containing the act D (0h), the cells were collected at 0h, 1h, 3h, 6h, 9h, 12h, and expression of the rest TUG1 in HK-2 cells were examined by RT-qPCR.

**Figure:**
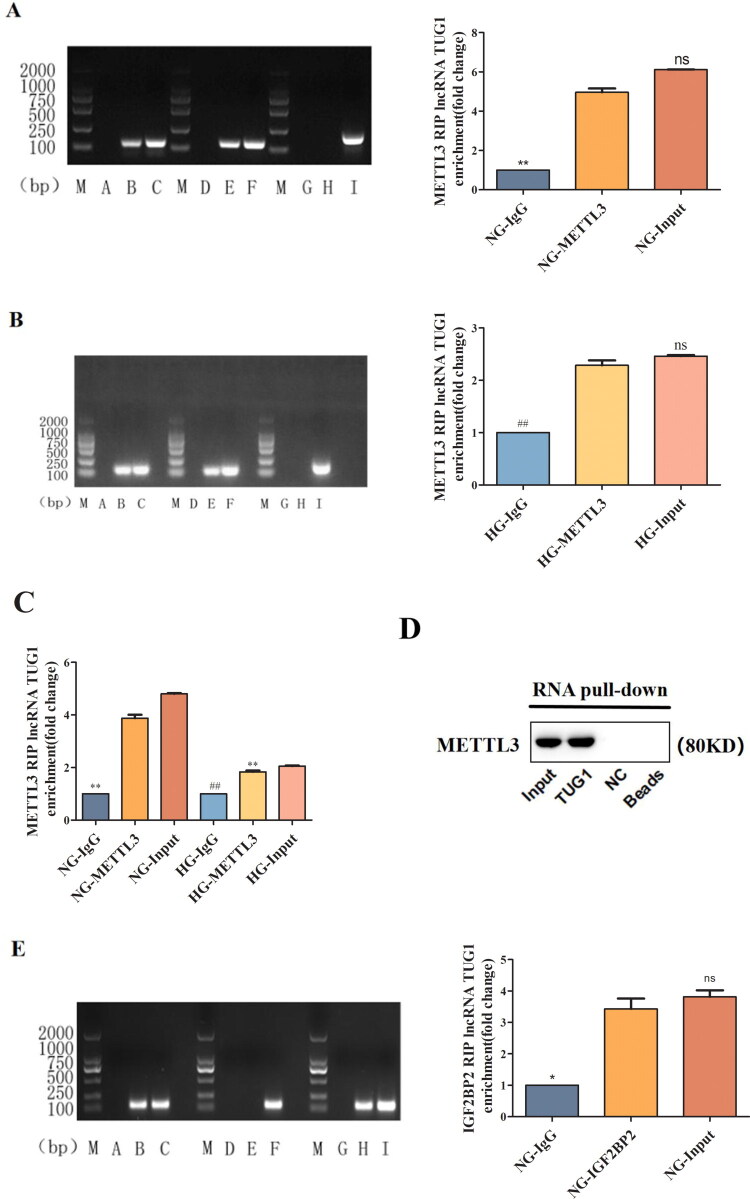

**Figure:**
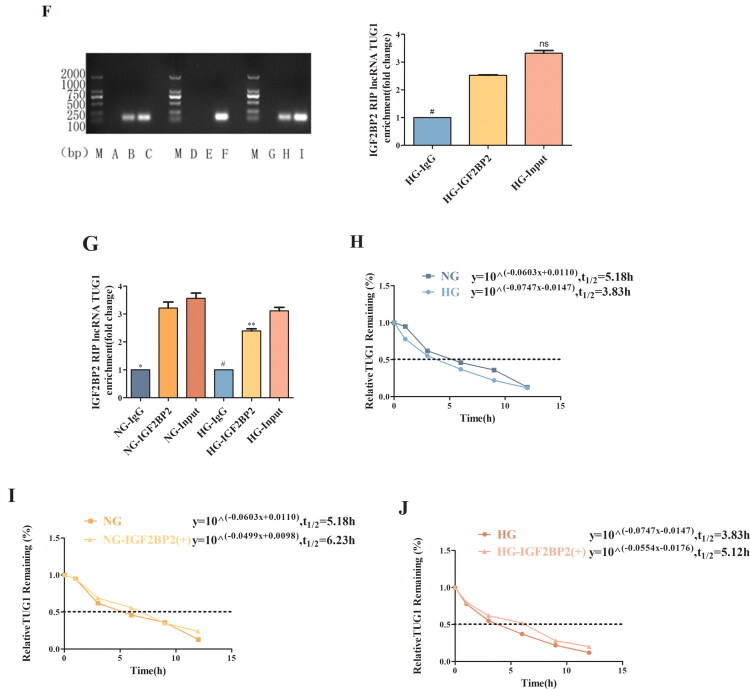

### 3.3. METTL3 overexpression increased m^6^A modification and TUG1 in HK-2 cells and db/db mice

To investigate the functional significance of METTL3 in DN, we transfected HK-2 cells with pcDNA3.1-METTL3-overexpressing vectors *in vitro*. Overexpression of METTL3 significantly increased the m^6^A modification level of total RNA (Figure 3(A)), the m^6^A modification level at sites 2047 and 4944 of TUG1 (Figure 3(B,C)), and the expression of TUG1 (Figure 3(D)), indicating that METTL3 can regulate TUG1 expression. To further verify the role of METTL3 *in vivo*, we conducted a preliminary experiment in 8-week-old db/m mice by injecting different concentrations of METTL3-overexpressing rAAV into the tail vein to verify its transfection efficiency and assess the viral toxicity. We also included a control + rAAV group to eliminate bias. Finally, we selected rAAV-METTL3 with the best transfection efficiency and the lowest viral toxicity at a concentration of 1 × 10^11^ GC/annual (Supplemental Figure 4A,B). The expression of GFP and obvious green fluorescence indicated successful virus injection (Supplemental Figure 5). METTL3 increased the level of m^6^A modification (Figure 3E) and the expression of TUG1 (Figure 3(F)), METTL3, and IGF2BP2 (Figure 3(G–J)) in db/db mice.

Figure 3.METTL3 Upregulated the level of the m^6^A modification and TUG1 in HK-2 cells and db/db mice. (A) The m^6^A content of total RNA in HK-2 cells after overexpression of METTL3 was examined by colorimetric method (*n* = 3). (B,C) the level of m^6^A modification of the two significant m^6^A sites of TUG1 after overexpression of METTL3 were detected by RNase MAZF-RT qPCR (*n* = 3). (D) Expression of TUG1 in HK-2 cells were examined by RT-qPCR (*n* = 3). (E) The m^6^A content of mice was examined by colorimetric method (*n* = 6). (F) TUG1 in kidneys and serum of mice was examined by RT-qPCR (*n* = 6). (G-J) METTL3 and IGF2BP2 in kidneys of mice were examined by Western blot, RT-qPCR and immunohistochemistry (*n* = 6). Data were presented as the mean ± SD. *p < 0.05, **p < 0.01, ***p < 0.001 vs. NG or db/m mice, #*p* < 0.05, ##*p* < 0.01, ###*p* < 0.001 vs. HG or db/db mice.

**Figure:**
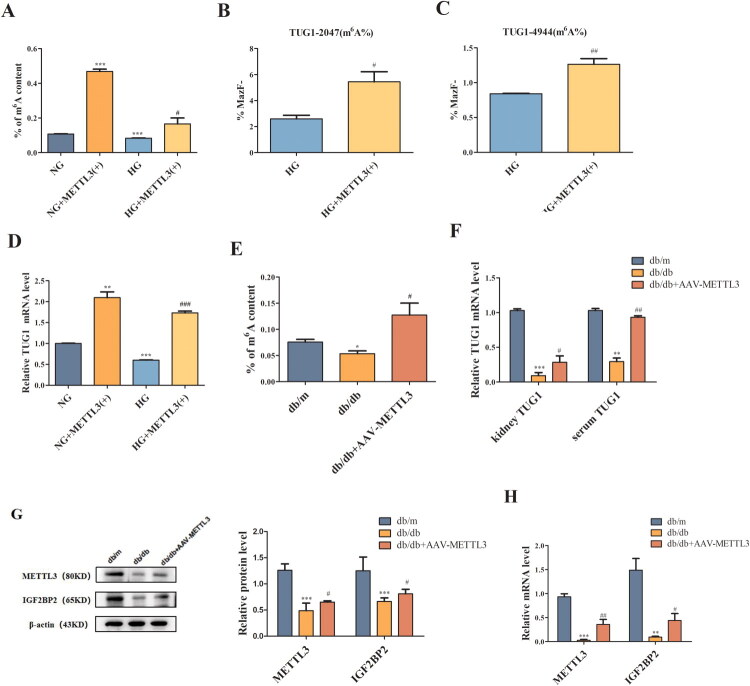

**Figure:**
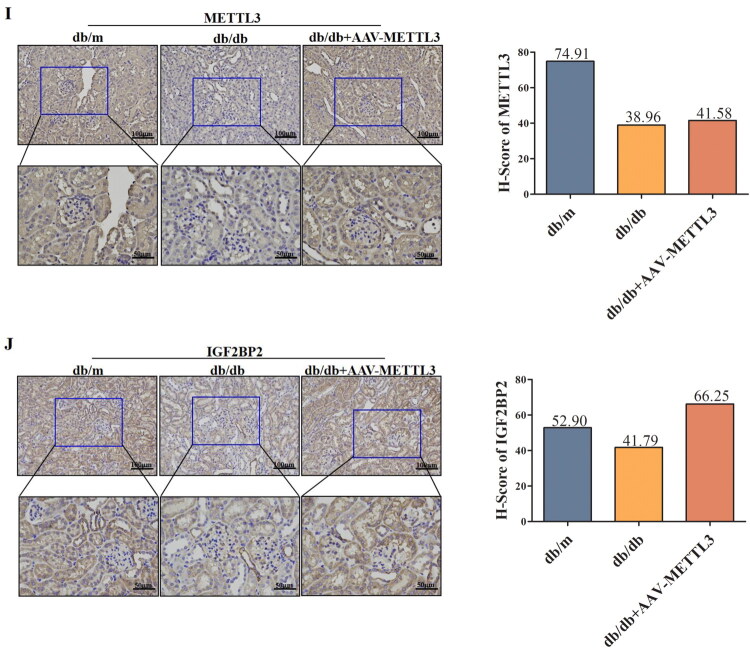


### 3.4. METTL3 ameliorates mitochondrial dysfunction in HK-2 cells and db/db mice

Mitochondrial dysfunction has been increasingly recognized as a key mediator in DN. Thus, *in vitro* we explored whether the TUG1/PGC-1α axis has a functional connection with METTL3 in HK-2 cells. We observed METTL3-mediated m^6^A modification of TUG1 promotes PGC-1α activation by enhancing promoter activity of PPARGC1A, which is significantly attenuated in siTUG1 transfected HK-2 cells. Thus verify the mechanistic link between METTL3-mediated m^6^A modification and TUG1’s regulation of PGC-1α (Figure 4(A)). Overexpression of METTL3 (Figure 4(B,C)) increased the expression of TUG1, PGC-1α and its downstream targets, including Nrf1, Nrf2, and TFAM (Figure 4(D–1)) in HK-2 cells; however, these effects were markedly abolished by transfection with siTUG1. METTL3 also rescued the mitochondrial dysfunction aggravated by HG and suppression of TUG1, as indicated by changes in mtDNA content (Figure 4(J)), ATP content (Figure 4(K)), mitochondrial respiratory chain complex I/III activity (Figure 4(L,M)); however, the activities of mitochondrial respiratory chain complexes II/IV were not significantly different (Figure 4(N,O)). The concentrations of ROS detected by MitoSOX Red (Figure 4(P)) and cell apoptosis (Figure 4(Q)) detected by flow cytometry notably increased in HG-induced and siTUG1-transfected HK-2 cells, which could be partially rescued by METTL3 overexpression. We also focused on alterations in mitochondrial morphology in HK-2 cells. Mitochondria exhibited severe swelling, vacuole formation, and mitochondrial crista fracture or loss in HG-induced and siTUG1-transfected HK-2 cells, which could also be partially rescued by METTL3 overexpression (Figure 4(R)). Overall, HG and siTUG1 aggravated mitochondrial dysfunction in HK-2 cells, and METTL3 partially reversed this effect. These data suggest that METTL3 promote PGC-1α transcription and alleviate mitochondrial dysfunction by targeting TUG1 in HG-induced HK-2 cells.

Figure 4.METTL3 Alleviated mitochondrial dysfunction by regulating the TUG1/PGC-1a axis in HK-2 cells. (A) Left, schematic of PPARGC1A promoter-luciferase constructs. Right, luciferase reporter activity in control and siTUG1 HK-2 cells. METTL3 promotes promoter activity, which is significantly attenuated in siTUG1 cells. ***p < 0.001. (B-D) Expression of METTL3 and TUG1 were examined by Western blot and RT-qPCR repectively in HK-2 cells. (E-I) Expression of PGC-1α, Nrf1, Nrf2 and TFAM were examined by Western blot and RT-qPCR repectively in HK-2 cells. (J) mtDNA content was examined by RT-qPCR. (K-O) ATP content, mitochondrial respiratory chain complex I, II, III and IV activities were examined according to the manufacturer’s instructions. (P) Use MitoSOX red to test the concentration of ROS under a fluorescence microscope. (Q) The degree of apoptosis under the different stimulations of HK-2 cells were evaluated through flow cytometry and Annexin-V-FITC/PI double staining. Q1 as necrotic cells, Q2 as advanced apoptotic cells, Q3 as complete live cells, Q4 as early apoptosis cells. (R) Observe mitochondria in HK-2 cells under TEM (amplification × 2000), and evaluate the ratio of damaged mitochondria to more intuitively demonstrate changes in mitochondria. Green arrow showing the normal mitochondria; red arrow showing the abnormal mitochondria. Data were presented as the mean ± SD (*n* = 6).*p < 0.05, **p < 0.01, ***p < 0.001 vs. NG, #*p* < 0.05, ##*p* < 0.01, ###*p* < 0.001 vs. HG, ※*p* < 0.05, ※※ *p* < 0.01, ※※※*p* < 0.001 vs. NG+METTL3(+), &*p* < 0.05, &&*p* < 0.01, &&&*p* < 0.001 vs. HG+METTL3(+).

**Figure:**
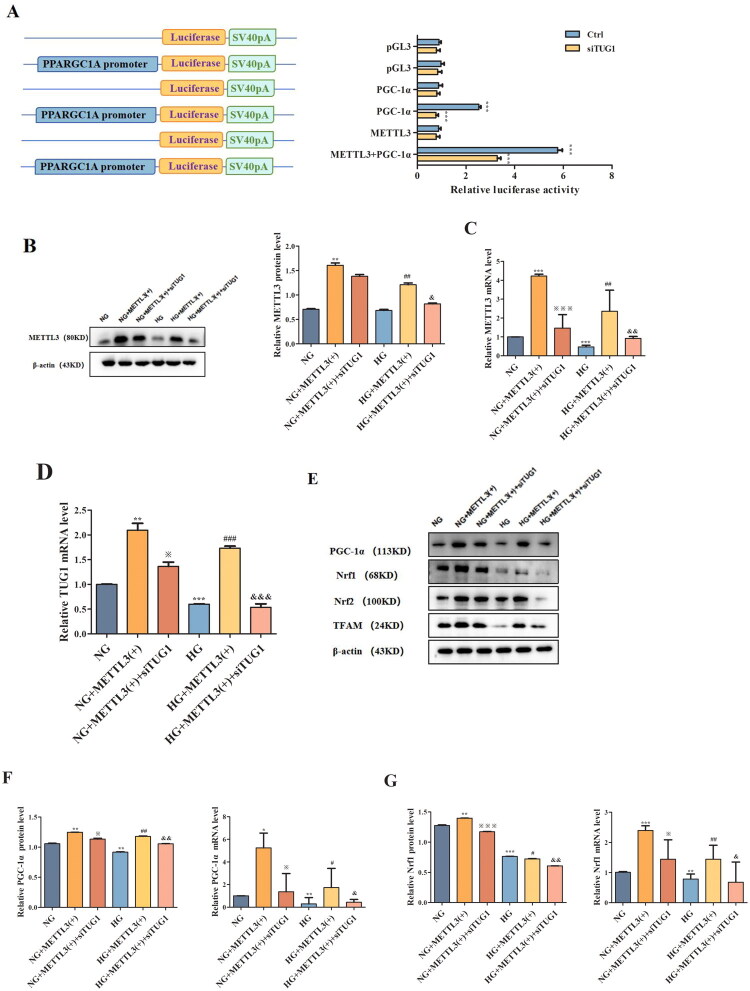

**Figure:**
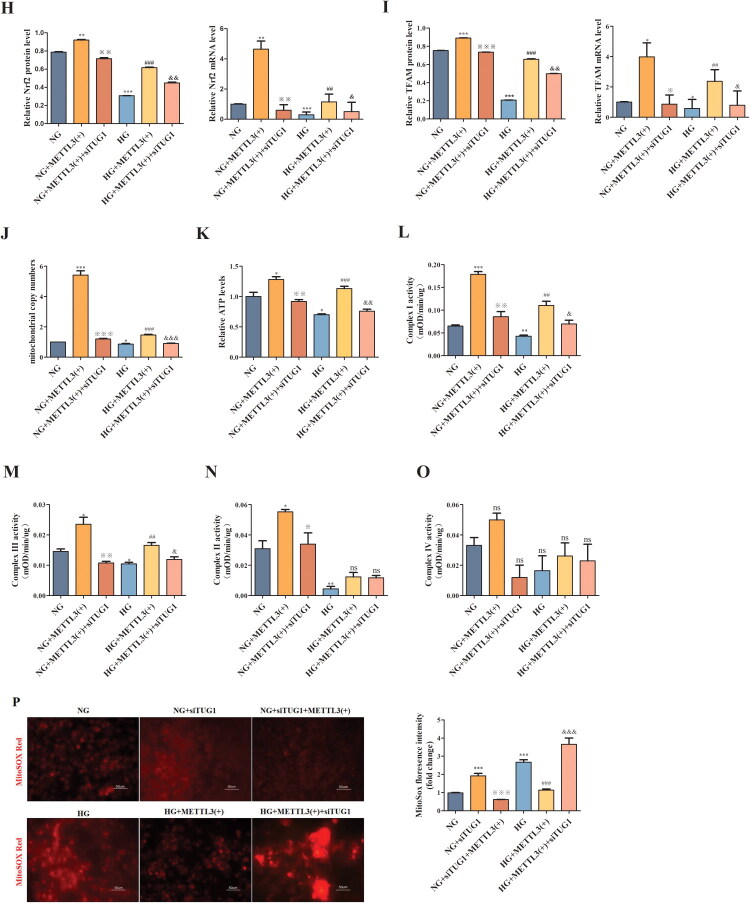

**Figure:**
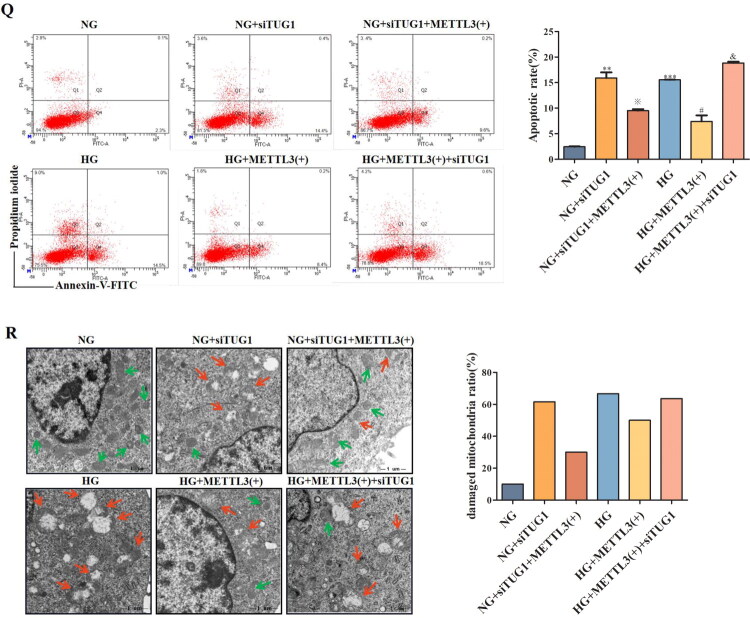

METTL3 can also rescue mitochondrial dysfunction in db/db mice. The levels of PGC-1α, Nrf1, Nrf2, and TFAM (Figure 5(A–F)), the content of mtDNA (Figure 5(G)) and ATP (Figure 5(H)), and the activities of mitochondrial respiratory chain complex I/III (Figure 5(I,J)) were significantly lower in the kidneys of db/db mice than in those of db/m mice; however, the activities of mitochondrial respiratory chain complexes II and IV (Figure 5(K,L)) were not significantly different. METTL3 overexpression can partially attenuate HG-induced suppression. Moreover, mitochondrial morphological damage and foot process fusion were more aggravated in the kidney tissues of db/db mice than in those of db/m mice. After rAAV-METTL3 overexpression, mitochondrial morphological damage and foot process lesions were alleviated (Figure 5(M)).

Figure 5.METTL3 Alleviated mitochondrial dysfunction in kidneys of db/db mice. (A–F) The expression levels of PGC-1, Nrf1, Nrf2 and TFAM of mice were examined by Western blot, RT-qPCR and immunohistochemistry respectively. (G) mtDNA content was examined by RT-qPCR. (H–L) ATP content, mitochondrial respiratory chain complex I, II, III and IV activities were examined according to the manufacturer’s instructions. (M) Observe mitochondria (amplification × 2000) and foot process (amplification × 1000) in kidneys of mice under TEM (amplification × 2000), and evaluate the ratio of damaged mitochondria to more intuitively demonstrate changes in mitochondria. The blue square showing mitochondrial morphological damage and foot process lesions. *p < 0.05, **p < 0.01, ***p < 0.001 vs. db/m mice, #*p* < 0.05, ##*p* < 0.01 vs. db/db mice.

**Figure:**
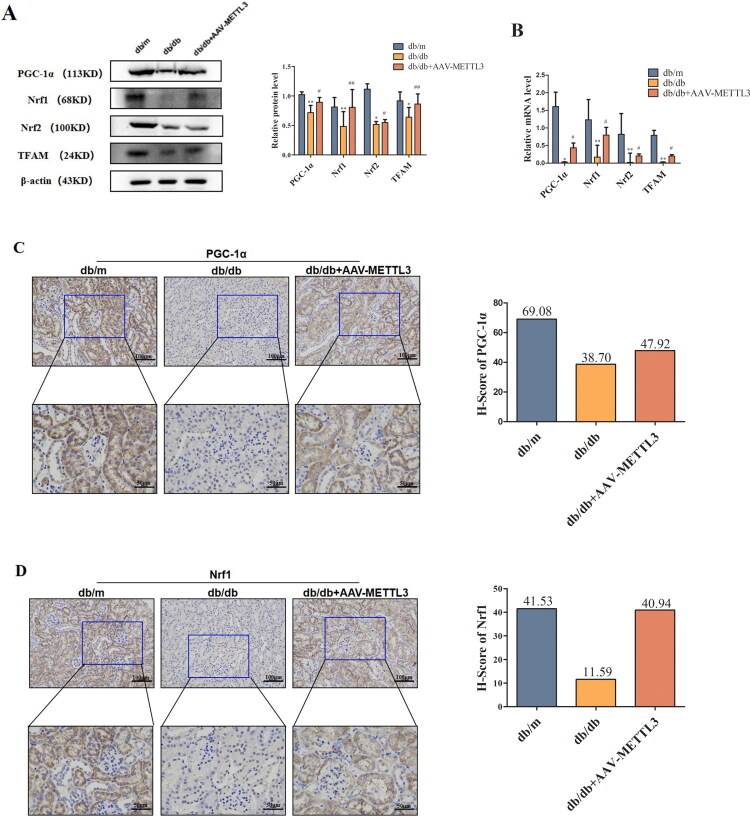

**Figure:**
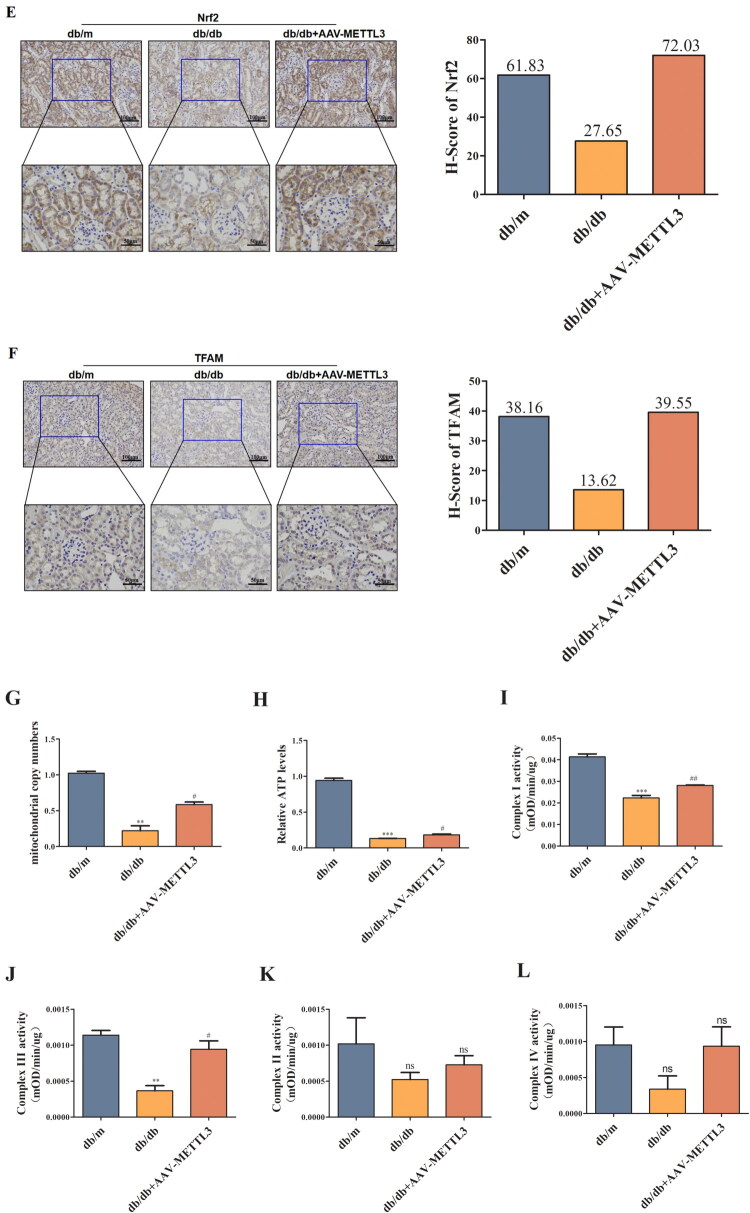

**Figure:**
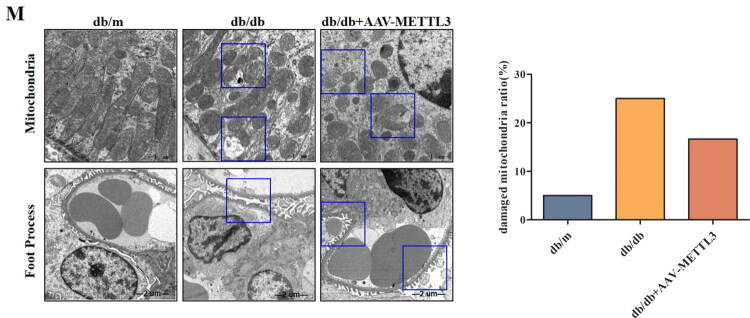

### 3.5. METTL3 overexpression alleviated kidney injury in db/db mice

**Figure 6.**
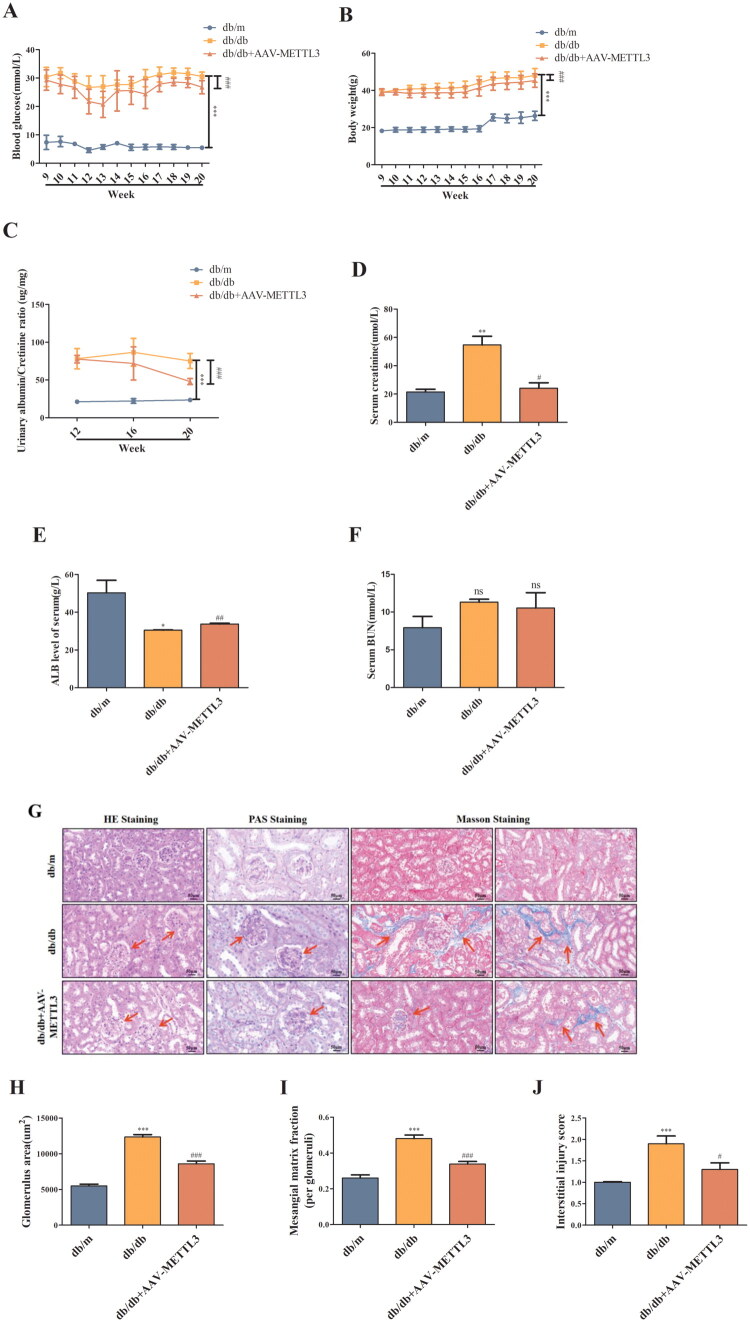
METTL3 Alleviated kidney injury in db/db mice. (A-B) Starting from 9-week-old mice, monitor the blood glucose and body weight of mice of each group (*n* = 6). (C-F) ELISA was used to detect ALB and creatinine in serum and urine, and BUN of mice (*n* = 6). (G-J) HE, PAS and masson staining were used to evaluate the damages of kidneys of mice (*n* = 6). Red arrow showing the abnormal morphological changes. Scale bar is 50 μm. *p < 0.05, **p < 0.01, ***p < 0.001 vs. db/m mice, #*p* < 0.05, ##*p* < 0.01, ###*p* < 0.001 vs. db/db mice.

Furthermore, injection of rAAV-METTL3 reduced the blood glucose level (Figure 6(A)), body weight (Figure 6(B)), urine albumin/creatinine ratio (Figure 6(C)), and serum creatinine level (Figure 6(D)) of db/db mice and increased the serum ALB level (Figure 6(E)), while the BUN level (Figure 6(F)) did not significantly differ. Moreover, HE staining revealed that the kidneys of db/db mice had enlarged and dilated glomeruli, glomerular hyaline degeneration, and tubular dilatation (Figure 6(G)). PAS staining revealed an increase in the glomerular mesangial matrix, expansion of the mesangial matrix, and an increase in the ratio of the matrix-positive area in the kidneys of db/db mice. Masson staining revealed an increase in the degree of tubulointerstitial fibrosis and the interstitial injury score, whereas METTL3 overexpression attenuated HG-induced renal pathological alterations in db/db mice (Figure 6(H–J)).

## 4. Discussion

With the development of second-generation sequencing technology, our knowledge of the structure and function of m^6^A has gradually improved [27]. Similar to DNA methylation, RNA m^6^A modification is a dynamic reversible modification. To date, researchers have identified 12 writers, 3 erasers, and 16 readers [28], but only METTL3, METTL14, WTAP, and FTO are involved in regulating the RNA m^6^A modification in DKD [29]. METTL3 is a catalytic subunit and the earliest reported m^6^A methyltransferase [30]. Other components mostly serve as regulatory subunits or RBPs coordinating or enhancing METTL3 activity [30]. However, due to the different modification sites and m^6^A binding readers, the function of m^6^A modification, especially those of METTL3, remain complicated and controversial. Recently, Zheng et al. reported that the total level of m^6^A-methylated RNA was markedly elevated in HG-induced HK-2 cells [25]. Wang et al. revealed that METTL3 silencing inhibited the cell death and inflammatory reaction in DN [31]. Chen et al. found that silencing of METTL3 prevented proliferation, migration, epithelial-mesenchymal transition (EMT), and fibrosis of HG induced HK-2 cells by decreasing WNT1-inducible-signaling pathway protein 1 (WISP1) with m^6^A modification pattern [32]. However, our findings showed that the total m^6^A modification level was markedly reduced and METTL3 alleviated renal tubular mitochondrial dysfunction by regulating the TUG1/PGC-1a axis in HG induced HK-2 cells. Similar changes were also observed in a previous study by Liu et al. who reported that in a mouse podocyte cell line (MPC-5) cultured in HG medium, METTL3 expression and m^6^A content decreased, and the total flavones of abelmoschus manihot (TFA) could ameliorate pyroptosis and injury by regulating phosphate and tension homology (PTEN)/phosphatidylinositol 3-kinases (PI3K)/Akt signaling in an METTL3-dependent manner [33]. Likewise, Tang et al. confirmed that METTL3 overexpression alleviated renal impairment and fibrosis in DN by enhancing Nuclear receptor-binding SET domain protein 2 (NSD2) mRNA stability [34]. Depending on the conditions, m^6^A exhibits tissue specificity, cell specificity, and temporal dynamics during aging [35], suggesting that the catalysis of m^6^A modification in response to HG conditions is complex and varies across different mouse, organisms, cell lines, and molecules, for immortalization of cell lines from different sources and independently bred animal with genetic drift over time can influence observed phenotypes by affecting differentiation potential, and maintaining or altering specific cellular functions and markers [36,37]. Additionally, different the concentration and intervention time of HG to HK-2 cells may cause the inconsistency of the results. Therefor, because of the error factor in experiments of cell and animal, total expression of m^6^A content and enzymes involved in m^6^A in certain animal strains and cell lines seem to gradually lose significance and persuasiveness, and expression of METTL3 in clinical samples of patients with biopsy confirmed DN seems more convincing. Moreover, as the enzymes involved in the dynamic regulation of the m^6^A methylation/demethylation imbalance *in vivo* and vitro are still debated in the research field, making the research significance of a specific RNA m^6^A modification and the effect of m^6^A regulating enzyme on RNA more important and meaningful. Thus, the mechanism of m^6^A RNA methylation needs further and deeper investigation.

Recent studies have shown that the new field of “RNA epigenetics” is booming, and m^6^A has been determined to be a posttranscriptional regulator of RNA species that can determine the destiny of cells [38,39]. In this study, we discovered that TUG1 significantly reduced the m^6^A modification level at only sites 2047 and 4944 in HG-cultured HK-2 cells, and METTL3 overexpression increased the expression of TUG1 and the m^6^A modification level at sites 2047 and 4944 of TUG1, indicating that not all m^6^A sites in TUG1 play specific roles and that the m^6^A modification at each m^6^A site may have different physiological functions. Specifically, METTL3 regulates the total RNA m^6^A modification level and the m^6^A modification level of TUG1. Also, we found that IGF2BP2 can bind to m^6^A-modified TUG1, extend its half-life, and increase its stability, consistent with the findings that IGF2BP2 can change RNA stability and degradation [17]. Therefore, METTL3 can promote the expression of TUG1 through the regulation of m^6^A modification in an IGF2BP2-dependent manner in HK-2 cells.

LncRNAs are critical to epigenetic regulation, and the regulation of various biological processes, including cell metabolism, proliferation, apoptosis, and differentiation [24,40,41]. Accumulating evidence shows that TUG1 plays a therapeutic role in many human kidney diseases, especially in the field of mitochondrial bioenergetics in DN [42]. For example, in mice mesangial cells (MCs), TUG1 can attenuate cell proliferation and ECM accumulation [43,44]. In NRK-52E cells, TUG1 can reduce cell fibrosis [45]. In mouse podocyte, TUG1 can attenuate ROS formation and cell apoptosis, improve podocyte foot process effacement, reduce glomerular basement membrane thickening and restore mitochondrial bioenergetics [42,46–48]. Thus different cell lines may affect TUG1 expression and the significance in DKD. In this study, we found that the level of TUG1 in HK-2 cells cultured under HG conditions and the kidney tissues and serum of db/db mice significantly decreased, and siTUG1 can aggravate mitochondrial dysfunction caused by HG, consistent with the findings of other studies and the recognized therapeutic effects of TUG1 in DN.

Mitochondrial dysfunction often occurs in the early stages of DN. Persistent hyperglycemia can affect the normal metabolism of renal tubular cells, and often occur earlier than structural changes in the glomerulus [49]. Due to the high demand for energy for solute reabsorption [50], the kidneys, particularly proximal tubular and medullary thick ascending limb cells, display a high mitochondrial density [51]. Tubular cells are very sensitive to kidney insults and require sufficient ATP to maintain their active transport of factors such as glucose and ions [52]. In this regard, when mitochondrial dysfunction occurs under persistent hyperglycemic conditions, oxidative phosphorylation and ATP levels decrease, and ROS are constantly generated, causing damage to mtDNA [52]. Therefore, mitochondrial dysfunction in renal tubules has gradually become a research focus in diabetic kidney damage. Long et al. reported that TUG1 interacts with its binding site in the upper area of the PPARGC1A promoter, enhancing the transcription of PPARGC1A mRNA, TUG1 also coactivates with PGC-1α [42], activating TFAM by interacting with Nrf1 and Nrf2 [53]. In mitochondria, TFAM and mtDNA combine to form a structure of a nuclear complex, which can protect mtDNA against ROS damage, thus maintaining the stability of mtDNA and protecting mitochondria [54]. We observed that HG stimulation inhibited the expression of PGC-1α and its downstream targets (Nrf1, Nrf2, and TFAM) *in vivo* and *in vitro*, as did reduce mtDNA content, ATP content, mitochondrial respiratory chain complex I/III activity, increase generation of ROS and accelerate cell apoptosis, presented as severe mitochondrial swelling, liquid foam formation, mitochondrial fracture, and sufficient fusion. While METTL3 overexpression reduced the inhibitory effect of HG on PGC-1α and its downstream targets through m^6^A modification of TUG1, thereby improving mitochondrial dysfunction in DN. Transmission electron microscopy of renal tubular epithelial cells in the renal cortex of db/db mice revealed mitochondrial swelling, liquid foam formation, mitochondrial fracture, reduction or loss and disruption of matrix density, partial fusion, and the disappearance of cracks. Therefore, the above mentioned data suggest METTL3 alleviates renal tubular mitochondrial dysfunction by regulating the TUG1/PGC-1a axis.

Meanwhile, we also found that the db/db mice exhibited pathological alterations in kidneys through HE, PAS, and Masson staining. However, METTL3 overexpression can ameliorate pathological damages to the kidney tissues of db/db mice, also significantly improved the serum creatinine, ALB levels and the urinary albumin/creatinine ratio in db/db mice, lowered the glucose level and slowed the increase in body weight of the db/db mice. Thus, these results suggest that renal function, insulin sensitivity, and fat content may be associated with METTL3, and more *in vitro* experiments on podocytes and mesangial cells are needed to investigate the role of METTL3 in diabetic nephropathy.

## 5. Conclusion

This study has once again affirmed that regulation of TUG1 is possibly implicated in the pathogenesis and development of DN. The m^6^A RNA methyltransferase METTL3 can enhance TUG1 mRNA stability and expression by IGF2BP2 to restore HG-induced renal tubular mitochondrial dysfunction, suggesting that upregulation of METTL3 or IGF2BP2 as possible upstream mechanisms for TUG1 may serve as candidate therapeutic strategies for mitochondria bioenergetics in DN through promoting trancription of PGC-1α. Since the upstream regulatory mechanisms of lncRNA TUG1 in DN are scarcely reported, more robust and further scientific experiments are needed to benefit this field.

## Supplementary Material

supplementary_tables.xlsx
